# *Schisandra chinensis* Stem Ameliorates 3-Nitropropionic Acid-Induced Striatal Toxicity via Activation of the Nrf2 Pathway and Inhibition of the MAPKs and NF-κB Pathways

**DOI:** 10.3389/fphar.2017.00673

**Published:** 2017-09-29

**Authors:** Eun-Jeong Kim, Minhee Jang, Min Jung Lee, Jong Hee Choi, Sung Joong Lee, Sun Kwang Kim, Dae Sik Jang, Ik-Hyun Cho

**Affiliations:** ^1^Department of Science in Korean Medicine, Graduate School, Kyung Hee University, Seoul, South Korea; ^2^Brain Korea 21 Plus Program, Graduate School, Kyung Hee University, Seoul, South Korea; ^3^Department of Convergence Medical Science, College of Korean Medicine, Kyung Hee University, Seoul, South Korea; ^4^Department of Cancer Preventive Material Development, Graduate School, Kyung Hee University, Seoul, South Korea; ^5^Department of Neuroscience and Physiology, Dental Research Institute, School of Dentistry, Seoul National University, Seoul, South Korea; ^6^Department of Physiology, College of Korean Medicine, Kyung Hee University, Seoul, South Korea; ^7^Department of Life and Nanopharmaceutical Sciences, College of Pharmacy, Kyung Hee University, Seoul, South Korea; ^8^Institute of Korean Medicine, College of Korean Medicine, Kyung Hee University, Seoul, South Korea

**Keywords:** stems of *Schisandra chinensis*, 3-nitropropionic acid, nuclear factor erythroid 2-related factor 2, mitogen-activated protein kinases, nuclear factor-kappa B

## Abstract

The beneficial value of the stems of *Schisandra chinensis* (SSC) in neurological diseases is unclear. We examined whether SSC aqueous extract (SSCE) alleviates striatal toxicity in a 3-nitropropionic acid (3-NPA)-induced mouse model of Huntington's disease (HD). SSCE (75, 150, or 300 mg/kg/day, p.o.) was given daily before or after 3-NPA treatment. Pre- and onset-treatment with SSCE displayed a significant protective effect and pretreatment was more effective as assessed by neurological scores and survival rate. These effects were related to reductions in mean lesion area, cell death, succinate dehydrogenase activity, microglial activation, and protein expression of inflammatory factors including interleukin (IL)−1β, IL-6, tumor necrosis factor-alpha, inducible nitric oxide synthase, and cyclooxygenase-2 in the striatum after 3-NPA treatment. Pretreatment with SSCE stimulated the nuclear factor erythroid 2-related factor 2 pathway and inhibited phosphorylation of the mitogen-activated protein kinase and nuclear factor-kappa B signaling pathways in the striatum after 3-NPA treatment. The gomisin A and schizandrin components of SSCE significantly reduced the neurological impairment and lethality induced by 3-NPA treatment. These results indicate for the first time that SSCE may effectively prevent 3-NPA-induced striatal toxicity during a wide therapeutic time window through anti-oxidative and anti-inflammatory activities. SSCE has potential value in preventive and therapeutic strategies for HD-like symptoms.

## Introduction

Huntington's disease (HD) is an inherited neurological disorder caused by abnormal expansion of a CAG (cytosine-adenine-guanine) repeat within exon 1 of the huntingtin (*Htt*) gene, located on chromosome 4 (Damiano et al., 2010; Ross and Tabrizi, 2011). The aggregation of mutant *Htt* protein leads to multiple issues, including toxic neuronal aggregates, transcriptional dysregulation, excitotoxicity, mitochondrial dysfunction with altered energy metabolism, and changes in axonal transport and synaptic dysfunction within the striatum and the cortex (Damiano et al., 2010; Ross and Tabrizi, 2011). HD is characterized clinically by subtle cognitive, motor, and psychiatric changes, which are collectively termed prodromal disease (Damiano et al., 2010; Ross and Tabrizi, 2011). Symptomatic treatments for abnormal motor functioning (mainly chorea), such as tetrabenazine (*Xenazine*®, the only US Food and Drug Administration-approved medication for HD) (Huntington Study Group, 2006) or neuroleptics (Barr et al., 1988) have limited benefits and are associated with disabling adverse effects, such as sedation/somnolence, insomnia, and depression. Development of efficient and safe drugs that forestall the outbreak or delay the onset of HD is critical.

Oxidative stress is considered a potential contributing factor to HD pathogenesis (Damiano et al., 2010; Ross and Tabrizi, 2011). Nuclear factor erythroid 2-related factor 2 (Nrf2), an important regulator of the antioxidative cellular response, interacts with antioxidant response element (ARE), and has a variety of cytoprotective roles against oxidative stress, apoptosis, and inflammation in the nervous system (Copple, 2012; Joshi and Johnson, 2012; Suzuki et al., 2013). Mice with Nrf2 deletions are inherently more susceptible to drug-induced toxicity and oxidative stress-induced diseases, including neurological diseases, while the overexpression of Nrf2 ameliorates the destructive effects of oxidative stress in various *in vivo* and *in vitro* disease models, including models of Parkinson's disease and HD (Copple, 2012; Joshi and Johnson, 2012; Suzuki et al., 2013). The Nrf2 signaling pathway is involved in the suppression of mitogen-activated protein kinases (MAPKs) and nuclear factor-κB (NF-κB), associated with inflammatory effects (Juge et al., 2007). The pathways are activated by pro-inflammatory cytokines, neurotrophic factors, neurotransmitters, neural injury, seizure activity, and proteins implicated in neurodegenerative disorders, including HD (Harper and Wilkie, 2003; Memet, 2006), and are upregulated by treatment with 3-nitropropionic acid (3-NPA) or kainate in the striatum, which mimics the pathology caused by mutant *Htt* (Sugino et al., 2000; Khoshnan et al., 2004). Although more studies are needed to fully identify the role of the Nrf2 and MAPKs/NF-κB pathways, it is reasonable to suggest that pharmacological modulation of these pathways may provide a new therapeutic target in HD.

*Schisandra (S.) chinensis (Turcz.) Baill* (*Omija* in Korean; *wǔ wèi zi* in Chinese; literally “five-flavor berry,” the common name) belongs to the genus *Schisandra* of the family Schisandraceae and is distributed and cultivated in northeastern China, far-eastern Russia, Japan, and Korea (Panossian and Wikman, 2008). The fruits of *S. chinensis* (FSC) have long been used in Oriental medicine to treat various diseases, such as gonorrhea, asthma, dysentery, enuresis, and dermatitis, and to relive excessive thirst (Panossian and Wikman, 2008). FSC extract and its constituents have gained attention for their potential role in the treatment of cardiovascular diseases like hypertension and myocardial infarction (Young Park et al., 2012; Chen et al., 2013), respiratory diseases including that caused by *Chlamydia pneumonia* and acute respiratory distress syndrome (Zhou et al., 2014), metabolic diseases like osteoporosis and diabetes (Kim et al., 2014), digestive diseases including hepatotoxicity (Wang, K. P. et al., 2014), and neurological diseases like ischemia (Jiang et al., 2014), which corroborates the observed effects of *S. chinensis* in traditional settings. These beneficial effects of *S. chinensis* are the result of anti-oxidant, anti-inflammatory, and anti-apoptotic activities related to the regulatory role of the Nrf2 and MAPKs/NF-κB pathways of chemical constituents including the lignans schizandrin, deoxyschisandrin (schizandrin A), gomisins, and pregomisin (Young Park et al., 2012; Chen et al., 2013; Jiang et al., 2014; Kim et al., 2014; Wang, K. P. et al., 2014; Zhou et al., 2014). The main constituents of FSC, including Schizandrin A/B/C and gomisin A/N/J, have been isolated from the stems of *S. chinensis* (SSC) (Lu and Chen, 2009; Zheng et al., 2014; Zhu et al., 2015). Schizandrins and gomisins have strong antioxidant and anti-inflammatory effects (Young Park et al., 2012; Chen et al., 2013; Jiang et al., 2014; Kim et al., 2014; Wang, K. P. et al., 2014; Zhou et al., 2014).

These findings strongly suggest the possibility of an essential role of SSC in physiological and pathological functions. However, to the best of our knowledge, there have been no reports on the beneficial effects of SSC. Herbal medicines including *S. chinensis* have traditionally been used as aqueous extract (Huh, 1613). Therefore, we investigated whether SSC aqueous extracts (SSCE) have a medicinal effect in a 3-NPA-induced striatal toxicity model. 3-NPA is an irreversible inhibitor of mitochondrial succinate dehydrogenase (SDH). It causes degeneration of striatal and cortical neurons in animals and results in gait abnormalities in an animal model of HD (Tunez et al., 2010; Mehrotra and Sandhir, 2014). We demonstrate that SSCE can be used as a beneficial tool to treat HD-like symptoms through the activation of Nrf2 signaling and inhibition of MAPKs and NF-κB signaling pathways.

## Materials and methods

### Animals and ethical approval

Adult male C57BL/6N mice (Narabiotec Co., Ltd., Seoul, Republic of Korea, 8–10 weeks of age, 22–25 g body weight were purchased from Taconic Biosciences Inc. (Hudson, NY, USA). The mice were housed at a constant temperature of 23 ± 2°C with a 12-h light-dark cycle (lights on from 08:00 to 20:00) and provided with food and water *ad libitum*. All experimental procedures were reviewed and approved by the Institutional Animal Care and Use Committee of Kyung Hee University. Proper randomization of laboratory animals and handling of data were performed in a blinded manner in accordance with recent recommendations from an NIH workshop on preclinical models of neurological diseases (Landis et al., 2012).

### Preparation of SSCE

**Figure 1:**
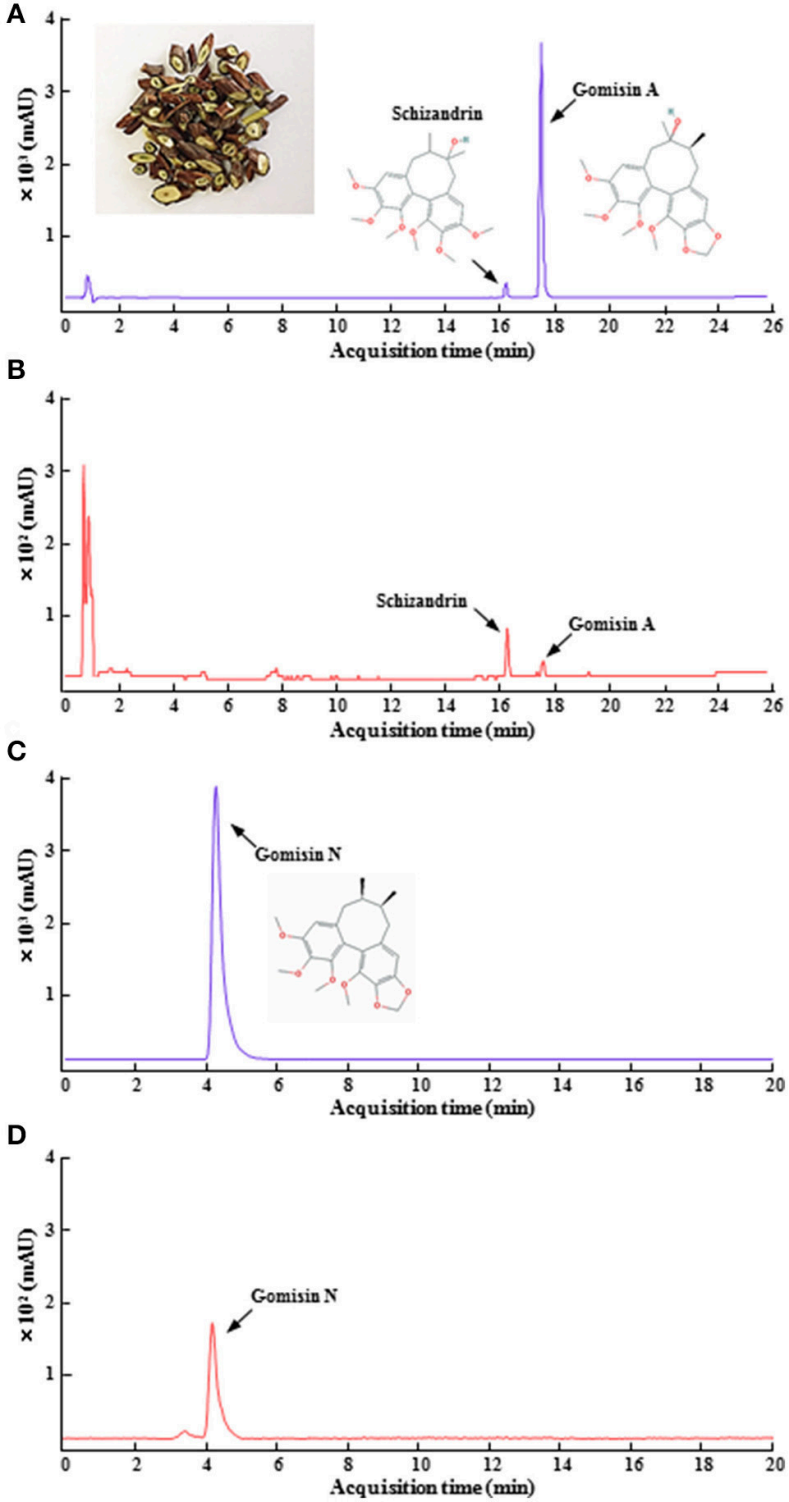
HPLC chromatogram of SSCE. Standard gomisin A, gomisin N, and schizandrin, the main components of SSCE **(A,C)** and their standardization **(B,D)** were analyzed using HPLC systems. The photo in **(A)** shows dried pieces of SSC. The chemical structures from the components are displayed in **(A,C)**.

SSC was obtained from Mungyeong-si Distribution Corporation (Mungyeong, Republic of Korea). The material was air-dried in a shaded lot and cut into small pieces (3–5 mm-thickness; Figure 1A). A voucher specimen (KHKM0030) was deposited at the Laboratory of Autoimmune and Neurodegenerative Diseases, Department of Convergence Science, College of Korean Medicine, Kyung Hee University. The SSC (200 g) was extracted with distilled water (2 L) for 1.5 h at room temperature, further extracted by continuous heating for 1.5 h under reflux conditions with hot water, and subsequently filtered by vacuum filtration. The filtrate was concentrated using a rotary vacuum evaporator (EYELAN-1200A, EYELA; Rikakikai Co. Ltd, Tokyo, Japan), lyophilized using a freeze drying system (OPR-FDA-8612; Operon Co. Ltd, Gimpo, Republic of Korea), and stored in a freezer (–80°C). The final yield of the SSCE was 10.55%.

### Quantitation of SSCE using ultraviolet high performance liquid chromatography (HPLC-UV)

Gomisin A, gomisin N, and Schizandrin were obtained from Wuhan ChemFaces Biochemical Co., Ltd. (Wuhan, China) and Sigma-Aldrich Co. (St. Louis, MO, USA). Quantitation of SSCE using HPLC-UV was accomplished by using a modification of a previously described protocol (Bae et al., 2012). Briefly, standard solutions containing each lignin were prepared in concentrations ranging from 5 to 250 ng/mL. One hundred milligrams SSCE was dissolved in 1 ml of distilled water. All standard and SSCE solutions were filtered through a 0.20 μm syringe filter (Merck Millipore Co., Schwalbach, Germany). The HPLC apparatus consisted of a model 1260 pump (Agilent Technologies, Santa Clara, CA, USA), a model G1367D autosampler, and a model G1315C DAD. Masshunter software (Agilent Technologies) was used. A YMC TRIART C18 column (50 × 2.0 ID-1.9 μm) was used for the HPLC analysis. The detection wavelength was set to 254 nm. The mobile phase contained acidified water with formic acid (0.1%, solvent A; Wako Pure Chemical Industries, Ltd., Osaka, Japan) and acidified acetonitrile (J.T. Baker Chemicals Co., Center Valley, NJ, USA) with formic acid (0.1%, solvent B). The gradient program consisted of 10% solvent B for 2 min, 50% solvent B for 20 min, and then was linearly increased to 80% solvent B for another 30 min. This linear gradient was followed by isocratic elution for 35 min and reconditioning steps to return to the initial mobile phase. The flow rate was 0.2 ml/min, and the injection volume of the standards and SSCE was 5 μL. A calibration curve was established using a methanol stock solution containing each dibenzocyclooctane lignan diluted to a specified concentration. The coefficient values (*r*^2^) were 0.999 and 1, demonstrating that the linearity in this range was sufficient to provide a highly accurate estimate of the content of gomisin A, gomisin N, and schizandrin in SSCE. Precision was determined using triplicate measurements of each standard, and the relative standard deviations (RSDs) were <2.2% (Figure 1).

### Experimental groups

The experiment was carried out in three stages. First, to determine the most effective dose and mechanism of administration of SSCE for pretreatment, mice were randomly divided into the following groups: sham (*n* = 10), 3-NPA (*n* = 16), 3-NPA + SSCE pretreatment (75, 150, and 300 mg/kg; *n* = 16 per each dose), and SSCE (*n* = 10). Second, to investigate the therapeutic time window of SSCE use, mice were randomly divided into sham (*n* = 4), 3-NPA (*n* = 9), 3-NPA + SSCE pre- (*n* = 9), onset- (*n* = 9), progression- (*n* = 9), and peak-treatment (*n* = 9), and SSCE (*n* = 4) groups. Third, to identify the active component(s) in SSCE, mice were randomly divided into sham (*n* = 4), 3-NPA (*n* = 7), 3-NPA + gomisin A, gomisin N, or schizandrin (*n* = 7 per each chemical), and gomisin A, gomisin N, or schizandrin alone (*n* = 4 per each chemical) groups. The group details are displayed in Table 1.

**Table 1:** Experimental protocols used for 3-NPA and SSCE treatment.

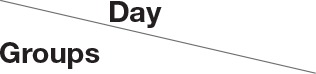		**–4**	**–3**	**–2**	**–1**	**0**	**0.5**	**1**	**1.5**	**2**	**2.5**	**3**	**4**	**5**	**6**	**7**	**8**
**3-NPA**						**1st**	**2nd**	**3rd**	**4th**								
SSCE	Pre-	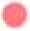	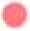	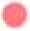	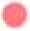	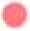		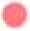		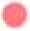							
	Onset-							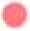		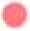							
	Progression-								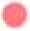		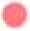						
	Peak-									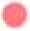		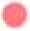	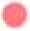	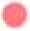	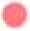	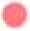	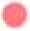
Components	Co-					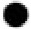		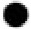		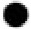							
Sacrifice and sampling										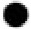	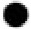						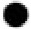

*3-NPA was treated i.p. twice daily for 2 days at 12-h intervals at a dose of 60 mg/kg on the first day and 80 mg/kg on the second day (60-60-80-80 dosing regimen). SSCE (75, 150, or 300 mg/kg) was administrated orally once daily from 4 days before 1st 3-NPA treatment (pre-), 12 h after 2nd 3-NPA treatment (onset-), 12 h after 3rd 3-NPA treatment (progression-), and 12 h after the last (4th) 3-NPA treatment (peak-). Components were treated i.p. once daily from 30 min before 1st 3-NPA treatment. Behavioral testing was conducted 24 h or 8 days after the last 3-NPA treatment. Striata were sampled 12 h after the last 3-NPA treatment for PCR analysis and 24 h after the last 3-NPA treatment for histopathological and immunoblotting analysis. 3-NPA, 3-nitropropionic acid; SSCE, stem of S. chinensis extract; pre-, onset-, progression-, and peak-, pretreatment, onset-treatment, progression-treatment, and peak-treatment, respectively*.

### Treatment with 3-NPA and SSCE

3-NPA was administered as previously described (Huang et al., 2006; Jang et al., 2013, 2014; Jang and Cho, 2016). Briefly, 3-NPA was dissolved in physiological saline to a concentration of 100 mg/ml (pH 7.4), passed through a 0.2 μm filter to remove any bacteria, and kept at –80°C until use. The 3-NPA solution was given intraperitoneally (i.p.) twice daily for 2 days at 12-h intervals (8:00 a.m. and 8:00 p.m.) at a dose of 60 mg/kg on the first day and 80 mg/kg on the second day (60–60–80–80 dose regimen). SSCE was prepared in physiological saline and administered at doses of 75, 150, or 300 mg/kg to determine the most effective dose. Subsequently, 300 mg/kg SSCE was given at the time of onset, progressive, and peak stages to investigate its therapeutic time window. Each experiment was repeated at least three times using the same protocol. Total daily dose of SSCE for mice was determined by formula for dose translation based on body surface area (Reagan-Shaw et al., 2008) after considering body weight of animals, final extract yield, and traditional dose in humans. Gomisin A (20 mg/kg), gomisin N (20 mg/kg), and schizandrin (45 mg/kg) were treated peritoneally once daily from 30 min before 3-NPA treatment.

### Neurological semi-quantitative assessment

To assess the severity of 3-NPA-induced neurological disorders, the previously described motor behavioral scale (Fernagut et al., 2002) and our standard for each subcriteria (Jang et al., 2014) were utilized. Briefly, the three-level (0, 1, and 2) scale was used to measure the severity of the following five items (maximal score = 10), which constituted the main motor symptoms observed: hindlimb clasping, global activity in a free-moving environment, hindlimb dystonia, truncal dystonia (kyphotic posture), and balance adjustment to a postural challenge. The neurological test was performed by an experimenter who was unaware of the experimental conditions and was done under constant temperature (23 ± 2°C) and humidity (55 ± 5%) in a quiet room, 24 h or 5 days after the final treatment.

### Histological assessment of striatal damage

We used our previous protocol to evaluate the histopathological changes in the striatum (Jang et al., 2013, 2014; Jang and Cho, 2016). Briefly, 24 h after the last 3-NPA treatment, the mice were anesthetized with ethyl ether and then perfused intracardially with saline and cold 4% paraformaldehyde in 0.1 M phosphate buffer (PB, pH 7.4). The brains (*n* = 5 in the sham group, *n* = 5 in the 3-NPA group, *n* = 7 in the 3-NPA + SSCE 75 mg/kg group, *n* = 7 in the 3-NPA + SSCE 150 mg/kg group, *n* = 7 in the 3-NPA + SSCE 300 mg/kg group, and *n* = 5 in the SSCE group) were immediately removed, post-fixed overnight in the same fixative at 4°C, and serially cryoprotected in 10, 20, and 30% sucrose in PBS for 48 h at 4°C. Sequential coronal sections (30 μm thickness) were acquired on a model CM3050S freezing microtome (Leica Biosystems, Wetzlar, Germany), starting from the anterior aspect of the corpus callosum and proceeding through the entire striatum (bregma 1.40–1.30 mm), according to the mouse brain atlas (Franklin and Paxinos, 2008). For histological assessment, every tenth section (at intervals of 300 μm) was processed for cresyl violet staining, dehydrated, and coverslipped with Permount (Fisher Scientific, Waltham, MA, USA). The stained sections (*n* = 3 per brain) from the level of the mid-striatum were captured using a DP70 image analysis system (Olympus, Tokyo, Japan) and the level of 3-NPA-induced striatal damage was analyzed using the NIH Image J program (http://rsb.info.nih.gov/ij/).

### Determination of SDH activity

A previously developed protocol (Jang et al., 2013, 2014; Jang and Cho, 2016) was used to investigate SDH activity in the striatum and the calf muscle. Briefly, fresh brains (*n* = 3 per group) were rapidly frozen in isopentane, sectioned at a thickness of 10 μm, and mounted on glass slides. The sections were incubated for 15 min in PBS at 37°C to activate the SDH. The three brain sections were washed in PBS and incubated in 0.05 M sodium succinate (Sigma-Aldrich), 0.3 mM nitroblue tetrazolium (Sigma-Aldrich), and 0.05 M PB (pH 7.6) for 20 min at 37°C. The sections were then rinsed in cold PBS for 5 min and dried at room temperature. Each section was digitally imaged using a DP70 image analysis system (Olympus) for quantitative microscopy. Quantification was performed as described previously using the Image J program. SDH activity was quantified in the cerebral cortex, and the striatum as regions of interest (ROIs) delineated on a digitized image. Extraction of color in each section was taken as the saturation value, and this value was used to represent SDH activity (% of sham group).

### Immunohistochemical staining

Immunohistochemical analysis of striatal sections was performed as previously described (Jang et al., 2013, 2014; Jang and Cho, 2016). Briefly, brain sections (30 μm thick) from each group (*n* = 5–7 per group) were incubated with 3% hydrogen peroxide in PBS and washed in PBS. The sections (*n* = 3 per brain) were then blocked with a solution containing 5% normal goat or horse serum, 2% bovine serum albumin, 2% fetal bovine serum, and 0.1% Triton X-100 for 2 h at room temperature. The sections were incubated overnight at 4°C with either rabbit anti-ionized calcium-binding adapter molecule 1 (Iba-1; 1:2,000; WAKO, Osaka, Japan), rabbit anti-glial fibrillary acidic protein (GFAP; 1:5,000; DAKO, Carpinteria, CA, USA), or rabbit anti-cleaved caspase-3 (1:1,000; Cell Signaling Technology, Danvers, MA, USA). The sections were washed in PBS and then incubated with biotinylated rabbit/mouse IgG antibody (1:200; Vector Laboratories, Burlingame, CA, USA) for 1 h at room temperature. After rinsing, the sections were incubated with avidin–biotinylated horseradish peroxidase (HRP) complex (1:200; Vector Laboratories) for 1 h at room temperature and visualized with 3,3′-diamino-benzidine (DAB). The sections were rinsed, dehydrated, and cover-slipped with Permount.

### Immunoblot analysis

Western blot analysis was performed as previously described (Jang et al., 2013, 2014; Jang and Cho, 2016). Briefly, 24 h after the last injection of 3-NPA, mice (*n* = 3 per group) were anesthetized and the striatum was removed with lysis buffer (50 mM Tris–HCl, pH 7.5, 150 mM NaCl, 1% Triton X-100, 10% glycerol, and protease inhibitor mixture). A total of 30 μg of tissue lysate from each sample was resolved by 10% SDS-PAGE. The proteins were transferred to polyvinylidene fluoride membranes and the membranes were blocked with 5% nonfat dry milk in Tween 20-containing Tris-buffered saline (TBST; 20 mM Tris, pH 7.4, 0.1% Tween 20, and 150 mM NaCl). The membranes were probed overnight with primary antibodies at 4°C, followed by incubation with HRP-conjugated secondary antibody at room temperature for 1 h prior to enhanced chemiluminescence analysis (Amersham Pharmacia Biotech, Piscataway, NJ, USA) and exposure to X-ray film. The primary antibodies included mouse anti-β-III-tubulin and rabbit ant-iNOS (1:500; Sigma-Aldrich), rabbit anti-phospho (p)-c-JUN N-terminal kinase (JNK), p-extracellular signal-regulated kinase (ERK), p-p38, p-IκBα, p-NF-κB p65, and cleaved caspase-3 (1:1,000; Cell Signaling Technology), rabbit anti-interleukin (IL)-1β, IL-6, tumor necrosis factor (TNF)-α, and mouse anti-nicotinamide adenine dinucleotide phosphate dehydrogenase (quinone) 1 (NQO1; 1:1,000; Cell Signaling Technology), rabbit anti-Iba-1 (1:1,000; WAKO), rabbit anti-GFAP (1:1,000; DAKO), rabbit anti-Nrf2 (1:200; Santa Cruz Biotechnology, Santa Cruz, CA, USA), rabbit anti-cyclooxygenase-2 (COX-2; 1:500; BD Biosciences, San Jose, CA, USA), and mouse anti-heme oxygenase-1 (HO-1; 1:1,000; Enzo Life Sciences, Farmingdale, NY, USA) antibodies. For normalization of the antibody signal, the membranes were stripped and reprobed with antibodies against JNK, ERK1/2, p38 (1:2,000; Cell Signaling Technology), or glyceraldehyde-3-phosphate dehydrogenase (GAPDH; 1:5,000; Cell Signaling Technology). After Western blot was performed several times, the density of each band was converted to a numerical value using the Photoshop CS2 program (Adobe, San Jose, CA, USA) after subtracting background values from an area of film immediately adjacent to the stained band. Data are expressed as the ratio of p-JNK, p-ERK, p-p38, p-IκBα, p-NF-κB, cleaved-caspase-3, β-III-tubulin, Iba-1, GFAP, IL-1β, IL-6, TNF-α, iNOS, COX-2, Nrf2, HO-1, and NQO-1 against total JNK, ERK 1/2, p38, or GAPDH for each sample.

### Detection of apoptosis with the tunel assay

The fragmentation of DNA was examined using an ApopTag® Peroxidase *in situ* Apoptosis Detection Kit (S7100) (Millipore) according to the manufacturer's instructions. Briefly, brain sections from each mouse (*n* = 5 per group) were subjected to enzymatic digestion with 20 μg/ml proteinase K for 5 min, treated with 5% hydrogen peroxide for 20 min to exhaust endogenous peroxidase activity, and washed with PBS. They were then immersed in ApopTag® equilibration buffer for 10 min to label the 3′-OH ends of fragmented DNA and incubated with terminal deoxynucleotidyl transferase-mediated UTP nick end labeling (TUNEL) at 37°C for 1 h. After being washed with PBS, the sections were incubated with DAB to detect signs of apoptotic cell death.

### Toxicological evaluation of SSCE

To examine whether SSCE has a toxic effect when used over the long term in mice, SSCE (16, 80, 400, and 2,000 mg/kg/day) was orally administrated to normal, 9-week-old, male mice for 15 days. Body weight, food intake, and water intake were measured daily and serum was obtained at 24 h after the last administration of SSCE. The serum levels of alanine aminotransferase (ALT), aspartate aminotransferase (AST), and lactate dehydrogenase (LDH) were measured using enzymatic or ultraviolet assays with an ALT, AST, or LDH detection kit (Roche, Basel, Switzerland) according to the manufacturer's instructions with a Cobas 8,000 modular analyzer (Roche) using a previously described protocol (Jang et al., 2014; Choi et al., 2015). General histological structure was evaluated by hematoxylin & eosin (H&E) staining as previously described.

### Statistical analyses

Statistical analyses were performed using the SPSS 21.0 package (SPSS Inc, Chicago, IL, USA) for Windows. Two-sample comparisons were carried out using the Student's *t*-test and multiple comparisons were made using two-way ANOVA with Tukey's post hoc test. All data are presented as means ± S.E.M. and statistical difference was identified at the 5% level unless otherwise indicated.

## Results

### Effect of SSCE on neurological score and survival rate following 3-NPA-treatment

#### Protective effect of SSCE pretreatment

**Figure 2:**
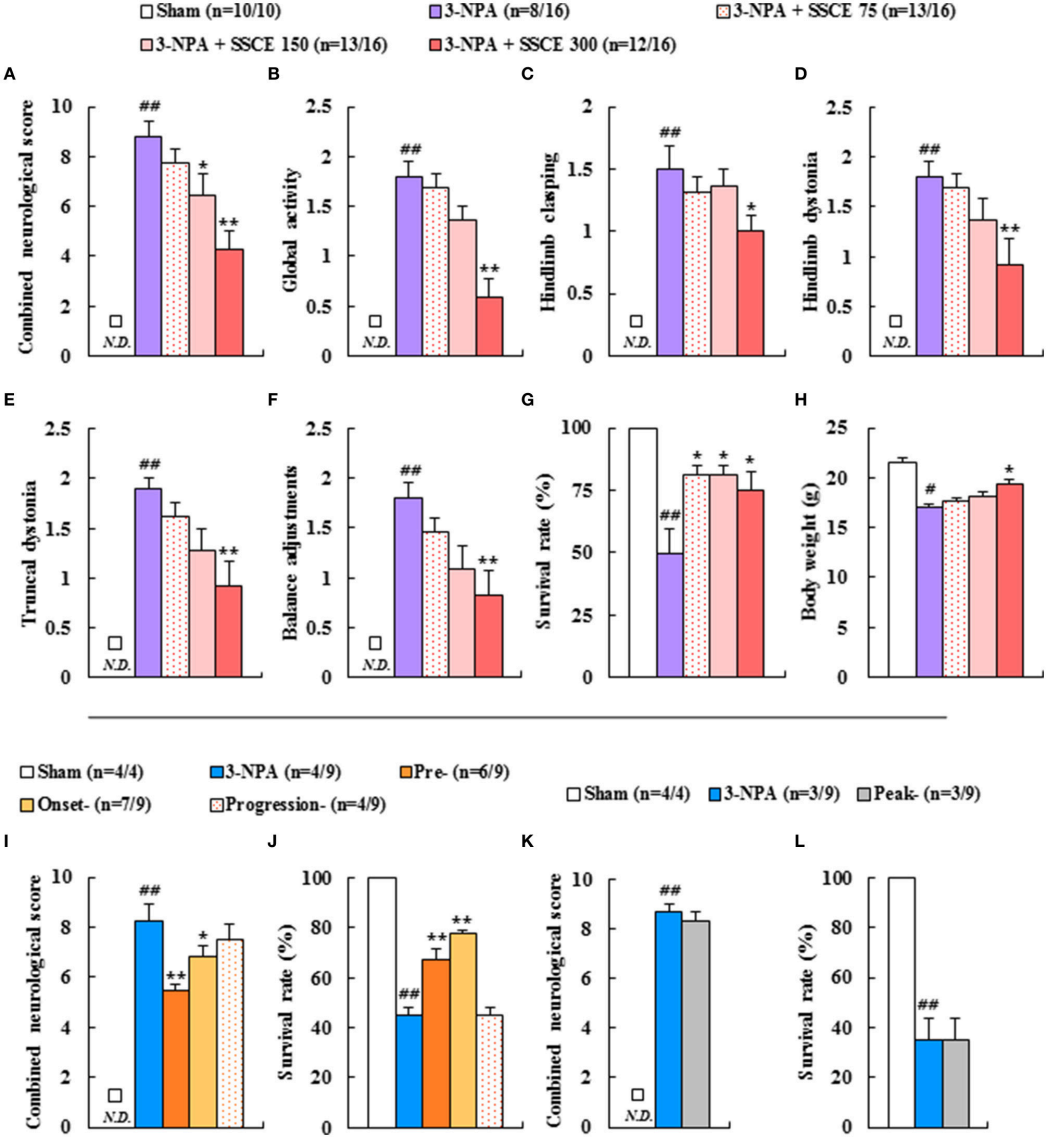
Effect of SSCE on neurological score, survival rate, and body weight after 3-NPA treatment. **(A–H)** SSCE (75, 150, or 300 mg/kg) was orally treated once daily from 4 days before 3-NPA treatment. At 24 h after the last (4th) 3-NPA treatment, the clinical signs of mice from each group (*n* = 10 or 16) were measured. The levels of global activity **(B)**, hindlimb clasping **(C)**, hindlimb dystonia **(D)**, truncal dystonia **(E)**, and balance adjustment to a postural challenge **(F)** were measured and their values were combined **(A)**. Survival rate **(G)** and body weight **(H)** were measured. **(I–L)** SSCE (300 mg/kg) was orally treated once daily from the onset (neurological score, 0–3), progression (neurological score, 3–6), and peak stages (neurological score, 6–10) of neurological symptoms of mice after 3-NPA treatment. At 24 h after the last 3-NPA treatment, the level of neurological impairment **(I,K)** and survival rate **(J,L)** were measured from each group (*n* = 4 or 9). *N.D*. in the graphs means not detected. The data are expressed as mean ± SEM. ANOVA testing was performed; ^##^*P* < 0.01 and ^#^*P* < 0.05 vs. sham group; ^**^P < 0.01 and ^*^P < 0.05 vs. 3-NPA group.

Qualitative determination of SSCE was performed using HPLC. Peaks for gomisin A, gomisin N, and schizandrin corresponded to standard at 18.096, 4.284, and 16.287 min, respectively. The final concentration for each constituent was 13.2, 19.8, and 532.2 ng/ml, respectively (Figure 1). To evaluate whether pretreatment with SSCE could alleviate 3-NPA-induced neurological signs, mice were treated with SSCE (75, 150, and 300 mg/kg/day) once daily from 1 h before each 3-NPA treatment. Twenty-four hours after the last (4th) injection of 3-NPA, mice from the 3-NPA group displayed symptoms of severe neurological deficits (combined neurological score, 8.8 ± 0.6; Figure 2A), including reduced global activity in a free-moving environment (1.8 ± 0.1; Figure 2B), hind limb clasping (1.5 ± 0.2; Figure 2C), hindlimb dystonia (1.8 ± 0.1; Figure 2D), truncal dystonia (kyphotic posture; 1.9 ± 0.1; Figure 2E), and balance adjustment to a postural challenge (1.8 ± 0.1; Figure 2F). Mice in the 3-NPA + SSCE group displayed slightly better neurological scores in a dose-dependent manner (combined neurological scores of 7.8 ± 0.6, 6.5 ± 0.9, and 4.3 ± 0.8 in the 75, 150, and 300 mg/kg/day, respectively), as compared to mice in the 3-NPA group (Figure 2A). More specifically, SSCE (300 mg/kg/day) significantly alleviated global activity (0.6 ± 0.2; Figure 2B), hindlimb clasping (1.0 ± 0.1; Figure 2C), hindlimb dystonia (0.9 ± 0.3; Figure 2D), truncal dystonia (0.9 ± 0.3; Figure 2E), and balance adjustment (0.8 ± 0.2; Figure 2F). Furthermore, the survival rate at the end of the experiment was increased by 81.3% (13/16), 81.3% (13/16), and 75% (12/16), respectively, in the 3-NPA + SSCE 75, 150, and 300 mg/kg/day SSCE groups, respectively, as compared to the 3-NPA group (50.0%, 8/16) (Figure 2G). The mean loss of body weight by 3-NPA (17 ± 0.43 g) was significantly ameliorated by SSCE treatment (19.4 ± 0.37 g) in the 300 mg/kg/day SSCE group (Figure 2H).

#### Therapeutic time window of SSCE

To determine the therapeutic time window of SSCE treatment against 3-NPA-induced neurotoxicity, mice were treated with SSCE (300 mg/kg/day; the most effective dose in Figures 2A–H) at various times during or after 3-NPA treatment. Groups were designated as pre-, onset-, progress-, and peak-treated groups. Based on the level of neurological impairment seen in Figures 2A–F and as we previously reported (Jang et al., 2014; Jang and Cho, 2016), we defined onset (initial stage of neurological score, 0–3), progression (middle stage of neurological score, 3–6), and peak stages (peak stage of neurological score, 6–10) of neurological symptoms for this experiment. The severity of neurological signs in the SSCE pre- (5.5 ± 0.2) and onset-treated groups (6.9 ± 0.8) were significantly lower than those in the 3-NPA group (8.3 ± 0.5). The survival rate in the 3-NPA group was 44.4% (n = 4/9) at the end of the experiment, but was increased in a time-dependent manner by SSCE pre- (66.6%, *n* = 6/9) and onset-treatment (77.7%, *n* = 7/9). However, progression- and peak-treatment with SSCE did not significantly improve neurological impairment or survival rate (Figures 2I–L). The results suggest that SSCE can reduce 3-NPA-induced neurological impairment.

### Protective effect of SSCE on striatal cell death induced by 3-NPA treatment

**Figure 3:**
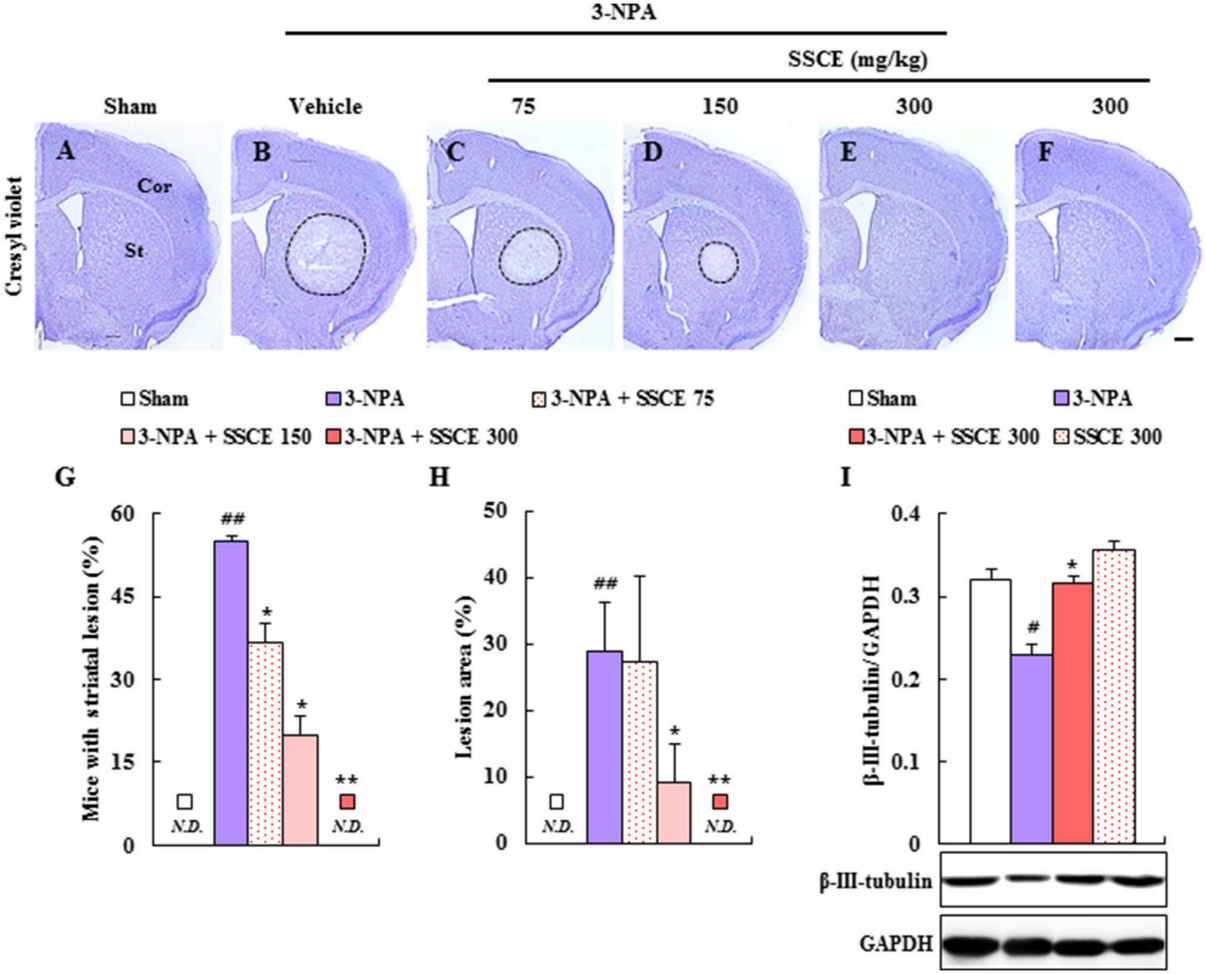
Effect of SSCE on striatal cell death after 3-NPA treatment. **(A–H)** Brain sections including striatum were obtained from each group at 24 h after the last (4th) 3-NPA treatment. The sections were stained using cresyl violet dye **(A–F)** and the ratio of mice with striatal lesion **(G)** and the level of striatal cell death **(H)** were quantified. **(I)** Lysate of the striatum obtained from each group at 24 h after the last 3-NPA treatment was analyzed for protein expression of β-III-tubulin by Western blot and quantified **(I)**. Cor, St, and dotted outline indicate the cortex, striatum, and striatal lesion, respectively. Scale bar = 100 μm. *N.D*. in the graphs means not detected. The data are expressed as mean ± SEM. ANOVA testing was performed; ^#^*P* < 0.05 and ^##^*P* < 0.01 vs. the sham group; ^*^P < 0.05 and ^**^P < 0.01 vs. the 3-NPA group.

3-NPA-induced neurological dysfunction results from neuronal death in the striatum (Fernagut et al., 2002; Jang et al., 2013, 2014; Jang and Cho, 2016). Appropriately, we investigated whether SSCE reduced striatal cell death. Twenty-four hours after the last (4th) 3-NPA treatment, brain slices including the striatum were stained with cresyl violet dye. Figure 3 shows representative striatal images from the sham, 3-NPA, and 3-NPA + SSCE (75, 150, and 300 mg/kg/day) groups. Fifty-five percent (*n* = 5/11) of the surviving 3-NPA-treated mice had visible bilateral striatal lesions (pale areas surrounded by dotted line), while the percentage of mice with striatal lesions was decreased to 36.3% (*n* = 4/11) and 20% (*n* = 2/10) in the groups pretreated with 75 and 150 mg/kg/day of SSCE, respectively (Figures 3A–G). Interestingly, visible striatal lesions were not detected in the group pretreated with 300 mg/kg/day of SSCE (0%; 0/10; Figures 3E,G). The ratio of the mean lesion area to the entire striatum was 28.7%, while the ratio was significantly reduced to 9.2% in the 150 mg/kg/day SSCE group (Figure 3H). Additionally, β-III-tubulin, a microtubule element of the tubulin family that is found almost exclusively in neurons, showed decreased expression in the striatum in the 3-NPA group (0.2 ± 0.01) compared with the striatum in the sham group (0.3 ± 0.01), whereas the decrease in expression was significantly rescued by pretreatment with SSCE (0.3 ± 0.01) (Figure 3I). The findings suggest that SSCE can reduce 3-NPA-induced striatal toxicity.

### Effect of SSCE on striatal SDH activity after 3-NPA treatment

**Figure 4:**
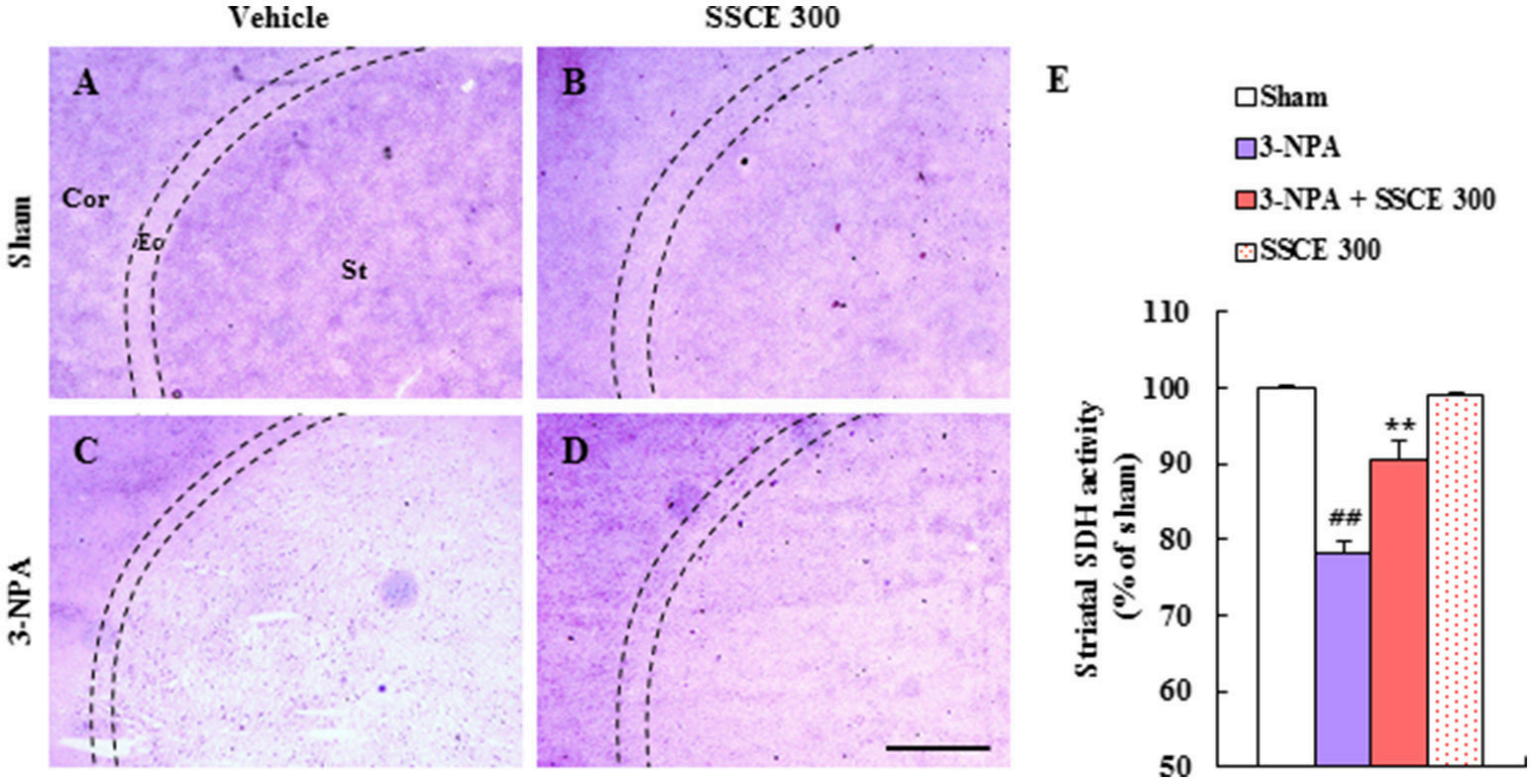
Effect of SSCE on striatal SDH activity after 3-NPA treatment. **(A–E)** Brain sections including striatum were obtained from each group at 24 h after the last (4th) 3-NPA treatment. The level of striatal SDH activity was analyzed histochemically **(A–D)** and quantified **(E)**. Quantified SDH activity is expressed as the percentage of striatum as compared with the sham group **(E)**. The Cor, St, and dotted line indicate the cortex, striatum, and external capsule (Ec), respectively. Scale bar = 100 μm. The data are expressed as mean ± SEM. ANOVA testing was performed; ^##^*P* < 0.01 vs. the sham group; ^**^P < 0.01 vs. the 3-NPA group.

3-NPA induces striatal neuronal cell death by inhibiting mitochondrial SDH activity (Fernagut et al., 2002; Jang et al., 2013, 2014; Jang and Cho, 2016). Appropriately, changes in SDH activity were examined by histochemical analysis in the striatum 24 h after the last (4th) 3-NPA treatment. Figure 4 shows representative striatal images from the sham, 3-NPA, 3-NPA + SSCE (300 mg/kg/day), and SSCE groups (Figures 4A–D). When the regional difference in SDH activity was semi-quantified, a decreased of 78.2 ± 1.6% was evident as compared to activity in striatum from sham group (100 ± 0.3%). However, SDH activity was significantly increased to 90.6 ± 2.4% by SSCE pretreatment (Figure 4E). SSCE itself did not significantly affect SDH activity in the striatum (99.1 ± 0.4%). The findings suggest that SSCE may alleviate 3-NPA-induced striatal toxicity by blocking the decline of SDH activity.

### Effect of SSCE on striatal apoptosis by 3-NPA treatment

**Figure 5:**
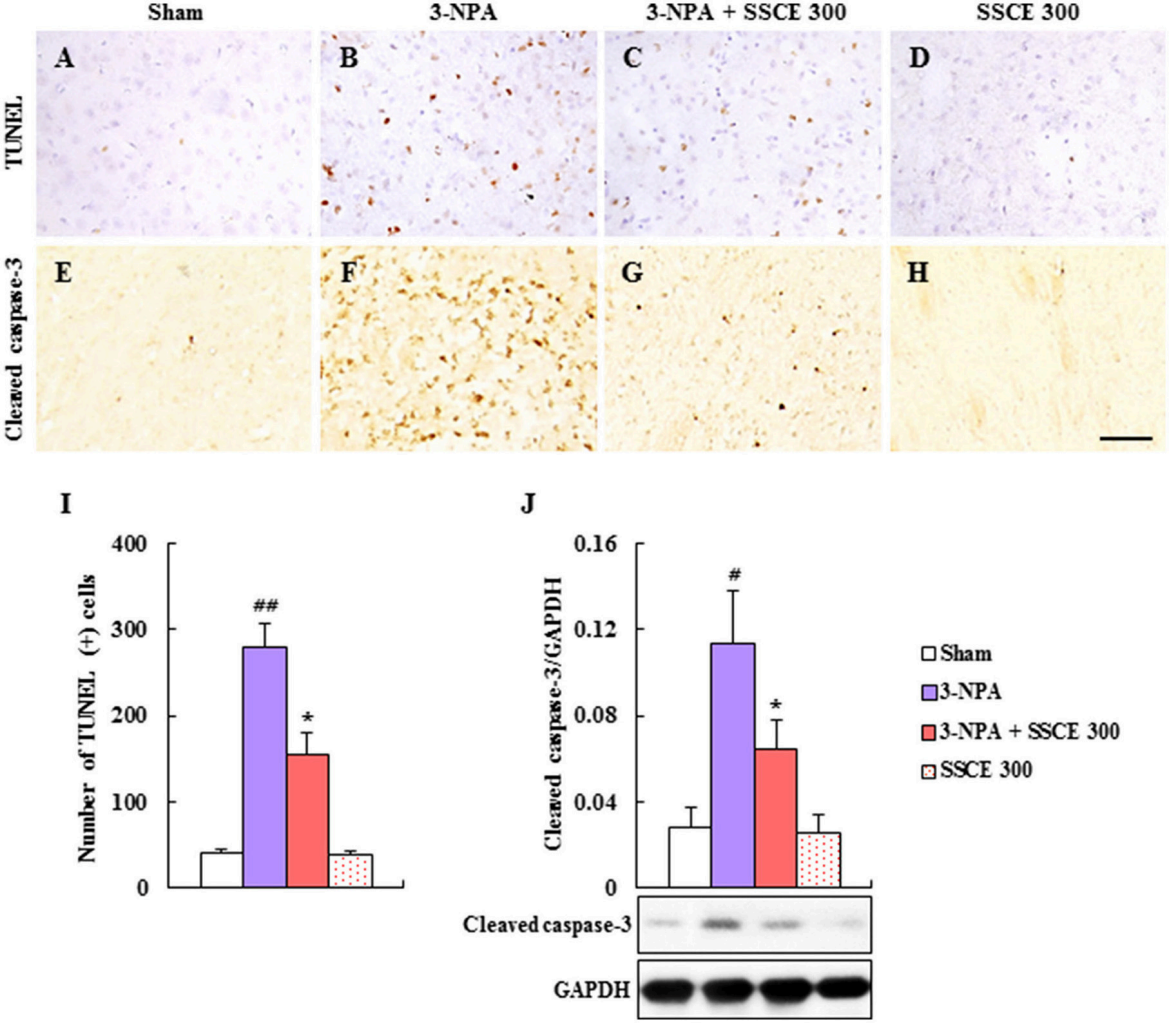
Effect of SSCE on the striatal apoptosis after 3-NPA treatment. **(A–I)** Brain sections including striatum were obtained from each group at 24 h after the last (4th) 3-NPA treatment. The brain sections were stained with a TUNEL kit **(A–D)** and immunostained with cleaved caspase-3 antibody **(E–H)**. Insets display high-magnification micrographs of the areas marked with squares **(A–D)**. Scale bars = 50 μm. The number of TUNEL-positive cells was quantified **(I)**. **(J)** Striatal tissue at 24 h after the last (4th) 3-NPA treatment was immunoblotted with cleaved caspase-3 antibody and the expression was quantified **(J)**. The data are expressed as mean ± SEM. ANOVA testing was performed; ^#^*P* < 0.05 and ^##^*P* < 0.01 vs. sham group; ^*^P < 0.05 vs. 3-NPA group.

Since the depletion of SDH activity in the striatum caused by 3-NPA leads to apoptosis in the striatum (Tunez et al., 2010; Jang et al., 2013, 2014; Jang and Cho, 2016), terminal deoxynucleotidyl transferase (TdT) dUTP nick-end labeling (TUNEL) staining (Figures 5A–D) and immunohistochemistry (Figures 5E–H) were used to determine whether SSCE reduces striatal apoptosis 24 h after the last (4th) 3-NPA treatment. The number of TUNEL-positive cells was significantly increased in the striatum in the 3-NPA group (Figures 5B,I) compared to the sham group (Figure 5A). But, the number of TUNEL-positive cells was significantly decreased in the 3-NPA + SSCE (300 mg/kg/day) group (Figures 5C,I). Also, the number of cleaved caspase-3-immunoreactive cells was increased in the striatum of the 3-NPA group (Figure 5F), as compared with the sham group (Figure 5E), while that number was significantly decreased in the 3-NPA + SSCE group (Figure 5G) as compared to the 3-NPA group, which corresponded to the expression pattern of cleaved caspase-3 protein as assessed by Western blot analysis (Figure 5J). SSCE itself did not induce apoptosis in the striatum as compared to the sham group. The results suggest that SSCE may reduce neurological dysfunction and striatal toxicity via inhibiting apoptosis in the striatum after 3-NPA treatment.

### Effect of SSCE on microglial activation in the striatum after 3-NPA treatment

**Figure 6:**
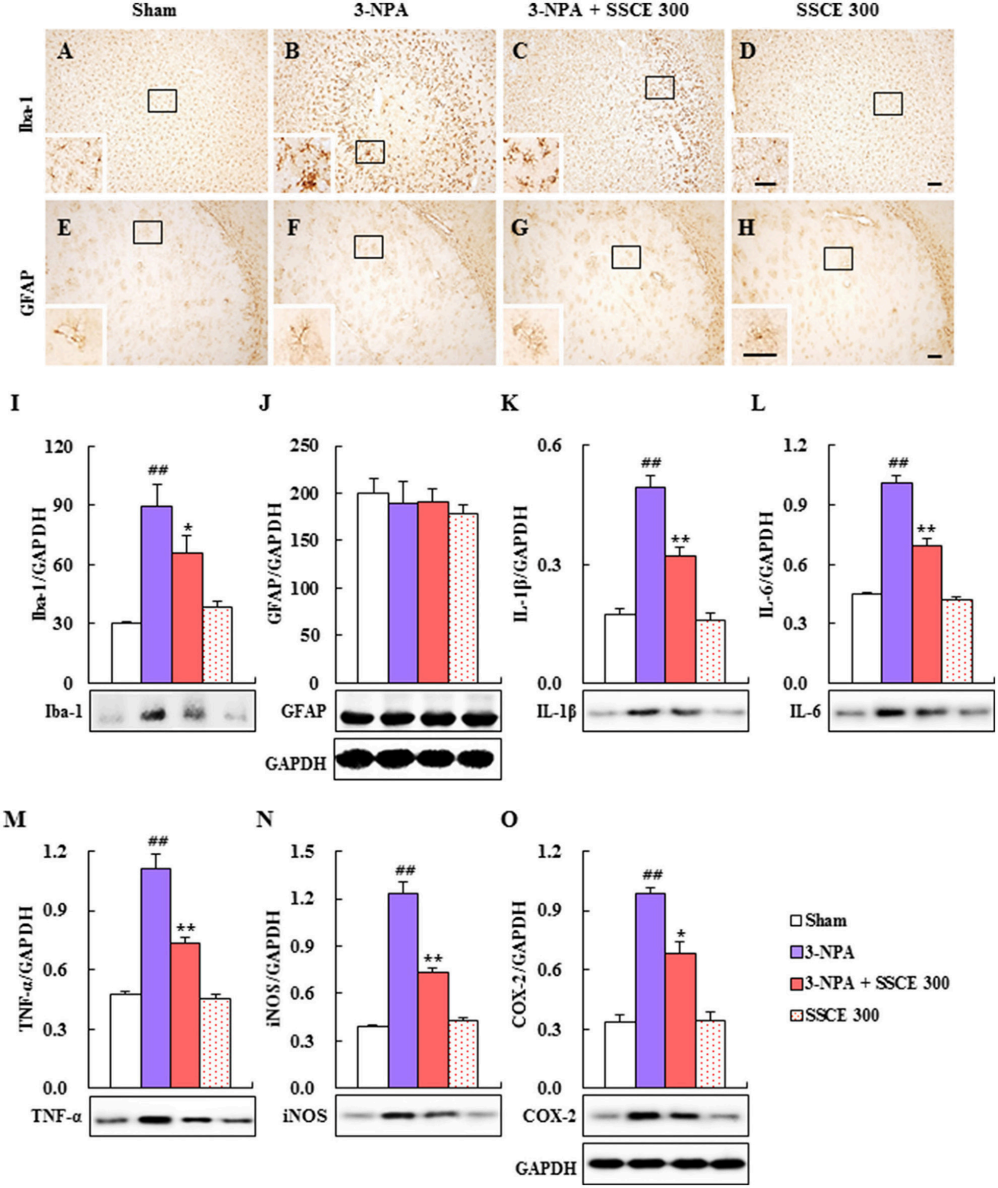
Effect of SSCE on microglial activation and activation of inflammatory mediators in the striatum after 3-NPA treatment. **(A–H)** Brain sections including striatum were obtained from each group 24 h after the last (4th) 3-NPA treatment. The brain sections were immunostained with anti-Iba-1 and-GFAP antibodies. The insets display high-magnification micrographs of the areas marked with squares. Scale bars = 100 μm. **(I–O)** Striatal tissues 24 h after the last 3-NPA treatment were analyzed by immunoblotting with antibodies against the Iba-1 **(I)**, GFAP **(J)**, IL-1β **(K)**, IL-6 **(L)**, TNF-α **(M)**, iNOS **(N)**, and COX-2 **(O)** and quantified **(I–O)**. GAPDH bands in **(J,O)** were shared in **(I,K–N)**, respectively. The data are expressed as mean ± SEM. ANOVA testing was performed; ^##^*P* < 0.01 vs. sham group; ^**^P < 0.01 and ^*^P < 0.05 vs. 3-NPA group.

Microglia are activated in neurological diseases including HD, and activated microglia release pro- and anti-inflammatory cytokines (Pavese et al., 2006; Lobsiger and Cleveland, 2007; Ferger et al., 2010). Therefore, we examined whether SSCE could inhibit microglial activation in striatal lesions after 3-NPA treatment (Figure 6). In striatal tissue from the 3-NPA group, Iba-1 (a marker for microglia/macrophage lineage cells)-immunoreactive cells; (Ferger et al., 2010; Jang et al., 2013, 2014; Jang and Cho, 2016) displayed activated morphology with enlarged cell bodies and short and thick processes (Figure 6B) compared to the sham group (Figure 6A), which generally showed the typical forms of resting cells including small cell bodies and thin processes. However, the level of activation of Iba-1-immunoreactive cells was reduced in the striatum in the 3-NPA + SSCE group (Figure 6C) compared to the 3-NPA group (Figure 6B), corresponding to a change in protein expression of Iba-1 as measured by immunoblot analysis (Figure 6I). Microglia were not activated by treatment only with SSCE (300 mg/kg) (Figures 6D,I). Astrocytes may be activated by the debris from dead cells or by activated microglia within or around neurodegenerative lesions (Lobsiger and Cleveland, 2007). However, in the current study, GFAP-immunoreactive astrocytes were not significantly affected within or around the striatal lesions 24 h after the last (4th) 3-NPA treatment compared with those in the sham and SSCE groups (Figures 6E–H), which reflects a lack of change in GFAP protein expression (Figure 6J). The findings suggest that SSCE inhibits microglial activation, but not astroglial activation, and is associated with a reduction in neurological impairment and striatal cell death.

### Effect of SSCE on inflammatory mediators in the striatum after 3-NPA treatment

Since activated microglia within or around lesions secrete factors that can be either beneficial or detrimental to neuronal survival (Pavese et al., 2006; Lobsiger and Cleveland, 2007; Ferger et al., 2010), we examined whether the inhibition of microglial activation induced by SSCE (Figures 6A–D,I) is closely connected with the down-regulation of inflammatory factors in the striatum. Immunoblot analysis determined that protein expression levels of IL-1β, IL-6, TNF-α, iNOS, and COX-2 were markedly increased by 0.49-, 1.01-, 1.10-, 1.23-, and 0.98-fold, respectively, in the striatum 24 h after the last (4th) 3-NPA treatment, while their expression levels were significantly decreased by 36, 32, 34, 41, and 31%, respectively, in striatal tissue from the 3-NPA + SSCE group (300 mg/kg/day) compared with the 3-NPA group (Figures 6K–O). These results suggest that SSCE can mitigate the neurological dysfunction and striatal toxicity induced by 3-NPA treatment by suppressing the expression of inflammatory modulators in the striatum.

### Effect of SSCE on oxidative stress in the striatum after 3-NPA treatment

**Figure 7:**
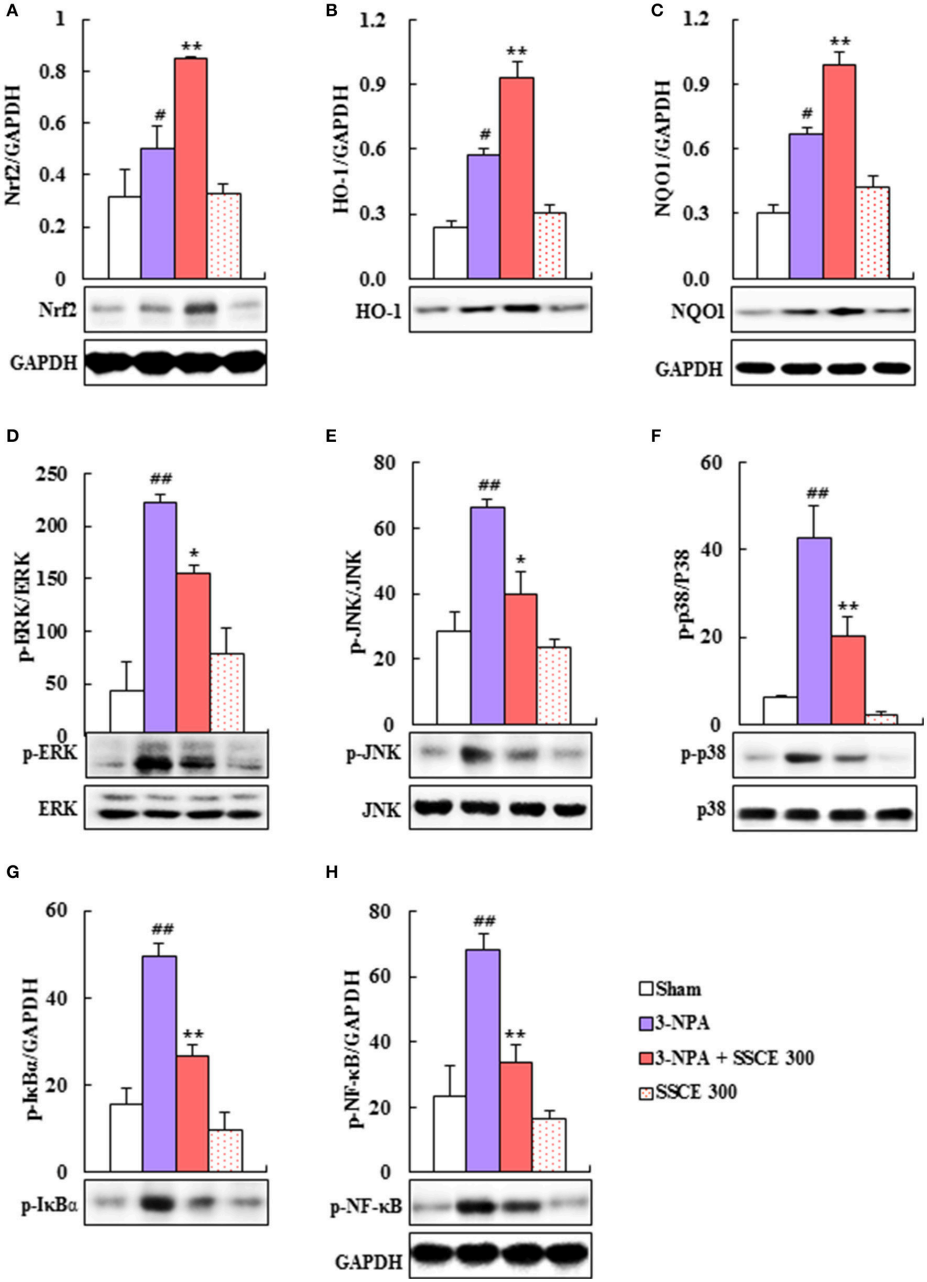
Effect of SSCE on Nrf2, MAPKs, and NF-κB pathways in the striatum after 3-NPA treatment. **(A–H)** Striatal tissues 24 and 12 h after the last 3-NPA treatment were analyzed by Western blotting **(A,D–H)** using Nrf2 **(A)**, HO-1 **(B)**, NQO-1 **(C)**, p-ERK **(D)**, p-JNK **(E)**, p-p38 **(F)**, p-IκBα **(G)**, and p-NF-κB **(H)** antibodies and quantified **(A–H)**. Bands of GAPDH in **(C,H)** were shared in **(B,G)**, respectively. ANOVA testing was performed; ^#^*P* < 0.01 and ^##^*P* < 0.01 vs. sham group; ^*^P < 0.05 and ^**^P < 0.01 vs. 3-NPA group.

*Schisandra chinensis* and its components have antioxidant and neuroprotective effects through Nrf2 transcriptional activation (Chen et al., 2008; He et al., 2014; Giridharan et al., 2015). Therefore, we investigated the effect of SSCE on the Nrf2 pathway. Nrf2, HO-1, and NQO-1 protein expressions were significantly increased (0.50-, 0.57-, and 0.67-fold, respectively) by 3-NPA treatment compared to expression in the sham group, whereas it was further increased (0.85-, 0.93-, and 0.99-fold, respectively) by SSCE (300 mg/kg) pretreatment compared to expression in the 3-NPA group (Figures 7A–C). Collectively, these results indicate that SSCE exerts its antioxidant activity through stimulation of the Nrf2 pathway in the striatum.

### Effect of SSCE on MAPKs and the NF-κB pathway in the striatum after 3-NPA treatment

Since MAPKs and the NF-κB pathway are activated in the striatum by 3-NPA treatment (Sugino et al., 2000; Khoshnan et al., 2004; Zheng et al., 2014), we tested the modulatory effect of SSCE on these pathways in the striatum after 3-NPA treatment. As expected, phosphorylation of ERK, JNK, and p38 proteins was significantly increased by 66.4-, 223.2-, and 42.9-fold, respectively, in the striatum 24 h after the last (4th) 3-NPA treatment, and by 59, 47, and 69%, respectively, compared with phosphorylation in the sham and SSCE alone groups, whereas the enhancement in activation was significantly reduced in the striatum in the 3-NPA + SSCE group (Figures 7D–F). We examined whether SSCE modulates the expression of NF-κB in the striatum after 3-NPA treatment. Protein expressions of p-NF-κB and p-IκBα were significantly increased by 68.0- and 49.4-fold, respectively, in the striatum 24 h after the final 3-NPA treatment, by 49 and 54%, respectively, compared to expression in the sham group, while the enhancement in activity was significantly decreased by SSCE treatment (300 mg/kg/day; Figures 7G,H). Activation of MAPKs or the NF-κB pathway was not increased or decreased by SSCE treatment alone (Figure 7). These results indicate that SSCE may reduce striatal toxicity and neurological dysfunction after 3-NPA treatment by suppressing MAPKs and the NF-κB pathways.

### Effects of representative components of SSCE on neurological function and lethality after 3-NPA treatment

**Figure 8:**
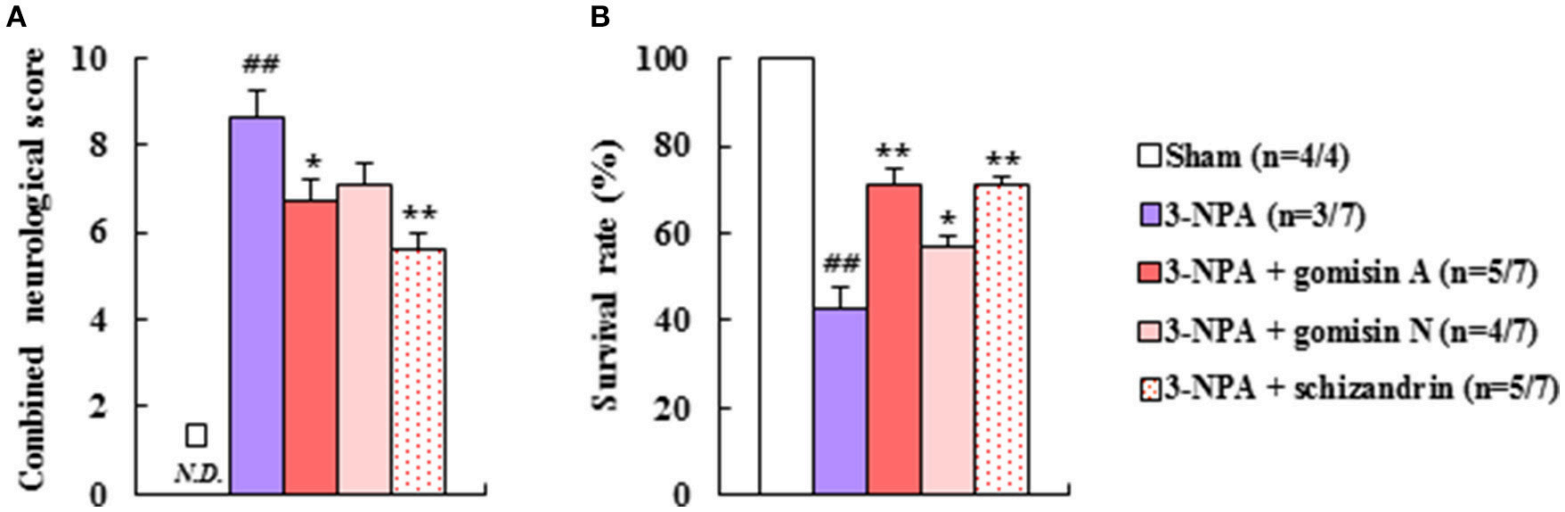
Effect of the main components of SSCE on neurological score and survival rate after 3-NPA treatment. **(A,B)** Gomisin A (20 mg/kg), gomisin N (20 mg/kg), and schizandrin (45 mg/kg) were treated peritoneally once daily from 30 min before 3-NPA treatment. The level of neurological impairment **(A)** and survival rate **(B)** were measured at 24 h after the last (4th) 3-NPA treatment. The data are expressed as mean ± SEM. ANOVA testing was performed; ^##^*P* < 0.01 vs. sham group; ^*^P < 0.05 and ^**^P < 0.01 vs. 3-NPA group.

To identify the active neuroprotective components in SSCE, we examined the effects of certain representative components (gomisin A, gomisin N, and schizandrin) on 3-NPA-treated mice. Pretreatment with gomisin A (20 mg/kg, i.p.) and schizandrin (45 mg/kg, i.p.) alleviated neurological impairment (6.8 ± 0.4 and 5.6 ± 0.4, respectively) and increased the survival rate (71.4% in both) 24 h after the final (4th) 3-NPA treatment compared to neurological impairment (8.7 ± 0.6) and survival rate (42.9%) in the 3-NPA group (Figures 8A,B). Although gomisin N (20 mg/kg, i.p.) did not significantly reduce neurological impairment (7.1 ± 0.5; *p* = 0.0542), increased survival rate (57.1%) (Figures 8A,B). These results indicate that gomisin A, gomisin N, and schizandrin may be the active components responsible for the beneficial effect of SSCE on 3-NPA-induced striatal toxicity.

### Administration of SSCE does not induce toxicity

Finally, to examine if SSCE has toxicity, we orally treated to normal male mice (9-weeks-old) with SSCE (16–2,000 mg/kg/day) for 15 days and assessed toxicity. Daily body weight (Supplementary Data 1A), daily feed intake (Supplementary Data 1B), daily water intake (Supplementary Data 1C), histological structure of liver (Supplementary Data 1D–H) and kidney (Supplementary Data 1I–M), and mean serum levels (AST, ALT, and LDH; Supplementary Data 1N–P) were not affected by administration of SSCE. Results indicate that prolonged administration of SSCE does not induce toxicity.

## Discussion

SSCE may alleviate 3-NPA-induced striatal toxicity by activating the Nrf2 pathway and inhibiting MAPKs and the NF-κB pathways. SSCE pretreatment alleviated neurological impairment, increased the survival rate, and reduced striatal cell death after 3-NPA treatment. Moreover, SSCE repressed SDH activity, apoptosis, microglial activation, and protein expression of IL-1β, IL-6, TNF-α, iNOS, and COX-2 in the striatum after 3-NPA treatment. SSCE stimulated the Nrf2 pathway and inhibited MAPK and NF-κB pathways. To the best of our knowledge, this is the first study to show that SSCE may attenuate 3-NPA-induced striatal toxicity by regulating various inflammatory cascades (Nrf2, MAPKs, and NF-κB pathways) and may be a potential therapeutic agent for HD-like symptoms.

The main ingredients of SSC, which include gomisin A/J/N and schisandrin (schizandrin) A/B/C, were also separated from the FSC (Lu and Chen, 2009; Zheng et al., 2014; Zhu et al., 2015). FSC extract and its constituents have beneficial effects in the treatment of various diseases including neurological diseases, such as cognitive and motor dysfunction (Panossian and Wikman, 2008), through anti-apoptotic (total extract and schisandrin B), anti-oxidative (total extract, schisandrin A, and schisandrin B), synaptic transmission (deoxyschizandrin), and anti-neuroinflammatory activities (schisandrin B and gomisin A) (Chen et al., 2008; Fu et al., 2008; He et al., 2014; Wang, X. et al., 2014; Giridharan et al., 2015; Zhang et al., 2015), which confirms the effects of FSC that have been reported by practitioners of traditional medicine (Huh, 1613). SSC also includes anwuweizic acid, daucosterol, (+)-deoxyschizandrin, (–)-dihydroguaiaretic acid, gomisin N, henrischinin A-C, schicagenin A-C, schinchinenin A-H, schinchinenlactone A-C, γ-schizandrin, β-sitosterol, tetradecanoic acid, and wuweizisu C (Shi et al., 2011; Song et al., 2013; Zheng et al., 2014). Anwuweizic acid, schicagenin A-C, schinchinenin A-H, and schinchinenlactone A-C have not been identified from FSC and their functions have not been investigated. The collective results strongly suggest that SSC may have a similar effect to FSC or a more impressive effect on the pathophysiological mechanisms of CNS. However, to the best of our knowledge, the beneficial effects of SSC have not yet been reported. The present findings demonstrate that SSCE has a neuroprotective effect against 3-NPA-induced striatal toxicity through anti-apoptotic, anti-inflammatory, and anti-oxidant activities that are related to the activation Nrf2 pathway and the inhibition of the MAPKs and NF-κB pathways.

The primary mechanism of 3-NPA-induced striatal toxicity involves irreversible inhibition of SDH, a key enzyme located in the inner mitochondrial membrane that is responsible for the oxidation of succinate to fumarate. Thus, regulating SDH activity in the striatum may be a therapeutic treatment option for diseases related to mitochondrial dysfunction. The representative medicinal herbs that alleviate neurological symptoms and striatal cell death by significantly preventing 3-NPA-induced inhibition of SDH activity include *Ginkgo biloba* extract (Mahdy et al., 2011) and Korean red ginseng (Jang et al., 2013), the antidepressant compound sertraline (Kumar and Kumar, 2009), and ethyl pyruvate (Jang et al., 2014). In the present study, SSCE also significantly ameliorated impaired SDH activity in the striatum following 3-NPA treatment, which was associated with improvement in behavioral impairment and beneficial mechanisms that included reduced apoptosis, inflammation, and oxidantion. Since impaired SDH activity can role as trigger of striatal cell death, maintenance of SDH activity by SSCE is an important finding.

We characterized the level of neurological impairment at various times to determine the therapeutic time window of SSCE in the 3-NPA-induced HD model. SSCE (300 mg/kg) applied prior to and following exposure to 3-NPA significantly alleviated neurological scores and lethality. However, progression- and peak-treatment with SSCE did not significantly improve neurological impairment or survival rate. This suggests that SSCE has preventive and therapeutic effects in the early stages of striatal toxicity caused by 3-NPA treatment and that its positive efficacy is related to the diminution of apoptosis via SDH inhibition, delayed cell death (secondary apoptosis) induced by inhibition of microglial activation and upregulation of pro-inflammatory cytokines, and anti-oxidation involving stimulation of the Nrf2 pathway in the striatum after 3-NPA treatment. This scenario is supported by the findings that 3-NPA induces secondary apoptosis through the generation of superoxide radicals after SDH inactivity (Dedeoglu et al., 2002) and that 3-NPA induces delayed cell death by activated microglial products surrounding the cells that die first (Brouillet, 2014).

Microglia are recruited and activated around or within neurodegenerative lesions earlier than astrocytes. Activated microglia release compounds that can be either beneficial or harmful to neuronal survival (Lobsiger and Cleveland, 2007). Clinical and positron emission tomography studies have shown that the level of microglial activation increases in proportion to the severity of HD symptoms (Pavese et al., 2006). Therefore, controlling recruitment and activation of microglia is an attractive therapeutic strategy for neurological disorders including HD (Lobsiger and Cleveland, 2007). Results demonstrate that SSCE inhibits microglial activation and protein expressions of IL-1β, IL-6, TNF-α, iNOS, and COX-2 in the striatum after 3-NPA treatment. Although the anti-inflammatory effects of SSCE have not yet been fully investigated, gomisin A/J/N and schisandrin B/C exert anti-neuroinflammatory activity in lipopolysaccharide-induced microglia by inhibiting NF-κB or/and MAPKs pathways (Oh et al., 2010; Zeng et al., 2012; Wang, X. et al., 2014). The findings support the possibility that SSCE has potent anti-inflammatory activity against 3-NPA-induced neurotoxicity.

Antioxidants can be used in preventive and therapeutic strategies for neurological disorders because oxidative stress contributes to numerous neurodegenerative diseases, such as HD. The Nrf2 pathway is one of the major regulators of oxidative stress (Copple, 2012; Joshi and Johnson, 2012). Mice with Nrf2 deletions exhibit aggravated 3-NPA- and malonate-induced motor dysfunction, striatal SDH and LDH activity, and striatal lesions as compared with wild type mice. However, oral administration of the Nrf2 activator tert-butylhydroquinone attenuates 3-NPA-induced striatal toxicity in wild type mice but not in Nrf2 knockout mice (Tarozzi et al., 2013). Schizandrin C is a component of FSC and SSC. It significantly induces the expression of phase II detoxifying/antioxidant enzymes including HO-1 and NQO1, inhibits lipoteichoic acid-stimulated reactive oxygen species (ROS) production in microglia (Park et al., 2013), and ameliorates learning and memory deficits caused by Aβ-induced oxidative stress in mice (Mao et al., 2015). Schisandrin B also protects against solar irradiation-induced oxidative stress in rat skin tissue (Lam et al., 2011). In the present study, pretreatment with SSCE increased protein expression of Nrf2-dependent gene products (HO-1 and NQO1) by stimulating the Nrf2 pathway in the striatum after 3-NPA treatment (Figure 7). Our previous study demonstrated that pre-activation of the Nrf2 pathway by pretreatment with other Nrf2 activators (dimethyl fumarate and antioxidant response element inducer-3) reduces 3-NPA-induced neurological impairment and lethality (Jang and Cho, 2016). The collective findings indicate that SSCE may include an essential inducible factor that protects against complex II inhibitor (3-NPA)-mediated neurotoxicity.

JNK, ERK, and p38 MAPKs are phosphorylated in polyglutamine-expanded *Htt*-mediated neuronal toxicity (Khoshnan et al., 2004; Wild and Tabrizi, 2014), in the striatal neurons of YAC128 and R6/2 transgenic mice (Wild and Tabrizi, 2014), and in a 3-NPA-induced neurotoxicity model (Sugino et al., 2000; Jang et al., 2013, 2014; Jang and Cho, 2016). However, inhibition of the phosphorylation of ERK or p38 by intrathecal administration of an ERK inhibitor (PD98059) or p-p38 inhibitor (SB203580) alleviates 3-NPA-induced motor dysfunction and increases the survival rate in the 3-NPA-induced HD model (Jang et al., 2013). SSCE significantly decreased 3-NPA-induced phosphorylation of ERK, JNK, and p38 MAPKs in the striatum (Figure 7), which corresponded to improvements in neurological symptoms (Figure 2) and striatal cell death (Figure 3). Gomisin J, gomisin N, schisandrin A, schisandrin B, and schisandrin C reportedly exert anti-inflammatory effects by down-regulating the ERK, JNK, or p38 MAPKs signaling pathways in lipopolysaccharide-treated RAW 264.7 cells (Ci et al., 2010; Oh et al., 2010) and in a dextran sulfate sodium-induced acute colitis model (Liu et al., 2015). In addition, schisandrin B prevents doxorubicin-induced cardiac dysfunction by inhibiting the p38 MAPK pathway (Thandavarayan et al., 2015). Taken together, despite the relative lack of information on the efficacy and critical mechanisms of action of SSCE, our findings indicate that SSCE may be used to regulate the phosphorylation of MAPK pathways in the therapeutic approach to HD-like symptoms.

The activation of the NF-κB pathway is increased in striatal cells following 3-NPA treatment and in cultured cells expressing mutant *Htt*. Preventing the degradation of NF-κB inhibitors with a dominant-negative ubiquitin ligase β-transducin repeat-containing protein decreases the toxicity of mutant *Htt* in MSNs. Inhibition of IKK activity with an N-terminally truncated form of IKK gamma (γ) reduces mutant *Htt*-induced toxicity in MSNs (Khoshnan et al., 2004). Sulforaphane, ethyl pyruvate, and Korean red ginseng extract prevent mitochondrial dysfunction by inhibiting the NF-κB p65 pathway in the striatum following 3-NPA treatment (Jang et al., 2013, 2014; Jang and Cho, 2016). Furthermore, blocking the NF-κB pathway by intrathecal injection of IκB inhibitor (pyrrolidine dithiocarbamate) before 3-NPA intoxication decreases lethality in an animal model (Jang et al., 2014). Various strategies have been attempted to clarify the critical role of the NF-κB pathway in the pathogenesis of HD and to determine the therapeutic safety and efficacy of its modification in HD. In this study, we demonstrate for the first time that SSCE alleviates NF-κB activity (expression of p65 and p-IκBα), which agrees with the reduced microglial activation and decreased protein expression of IL-1β, IL-6, TNF-α, iNOS, and COX-2 in the striatum following 3-NPA treatment (Figure 6). Taken together, our findings suggest that SSCE might alleviate 3-NPA-induced striatal toxicity by inhibiting activation of the NF-κB pathway and that SSCE could be useful as a treatment for HD-like syndromes.

The main components of *S. chinensis*, including gomisin A, gomisin N, and schizandrin, have neuroprotective effects (Chen et al., 2008; Fu et al., 2008; Park et al., 2013; Jiang et al., 2014; Wang, X. et al., 2014; Giridharan et al., 2015; Mao et al., 2015; Zhang et al., 2015). In the present study, pretreatment with gomisin A (20 mg/kg, i.p.) and schizandrin (45 mg/kg, i.p.) significantly reduced neurological impairment and lethality compared to administration of 3-NPA alone. Although not statistically significant (*p* = 0.0542), gomisin N (20 mg/kg, i.p.) showed a tendency to reduce neurological impairment. And gomisin N significantly reduced lethality (Figure 8). These results suggest that certain components, including gomisin A, gomisin N, and schizandrin, may be active in the protective effects of SSCE on 3-NPA-induced neurotoxicity, and that the essential efficacy and cellular mechanisms of action of the active components of SSCE may be exploited as useful adjuvant therapies and in functional foods to benefit patients with neurological diseases, including HD.

The relationship between Nrf2 and NF-κB is not well characterized. However, the identification of NF-κB binding cites in the promoter region of the *Nrf2* gene suggests cross-talk between these two regulators of inflammatory processes. NF-κB signaling inhibits the Nrf2 pathway through the interaction of p65 and KEAP1 (Sandberg et al., 2014). Furthermore, the protective up-regulation of the Nrf2 pathway is mediated by activation of several kinases including JNK, ERK, and p38 (Sandberg et al., 2014). In the present study, SSCE activated the Nrf2 pathway (Nrf2, HO-1, and NQO-1) and inhibited activation of MAPK pathways (JNK, ERK, and p38) and NF-kB pathways (IkBα and NF-κB p65) in the striatum after 3-NPA treatment (Figure 7). Although further studies are necessary to demonstrate the details of the mechanism, our findings suggest that SSCE may exert a neuroprotective effect by regulating cross-talk between Nrf2, MAPKs, and NF-κB pathways.

## Conclusions

The most appropriate therapeutic strategy for the treatment of HD is currently unclear. Herein, we demonstrate for the first time the potential neuroprotective value of SSCE for the treatment of HD-like symptoms. Pre- and onset-treatment with SSCE improved neurological scores and lethality after 3-NPA treatment, but the same positive effects were not observed following progression- and peak-treatment. Pretreatment with SSCE significantly reduced SDH activity, apoptosis, microglial activation, and protein expression of IL-1β, IL-6, TNF-α, iNOS, and COX-2 in the striatum after 3-NPA treatment, corresponding to the activation of the Nrf2 pathway and attenuation of MAPKs and NF-κB pathways in the striatum following 3-NPA treatment. Additionally, gomisin A, and schizandrin treatment significantly improved neurological impairment and lethality compared to treatment with 3-NPA alone. These findings strongly indicate that SSCE has beneficial effects against 3-NPA-induced striatal toxicity that are due to anti-oxidative and anti-inflammatory activities. Further studies including those that address the molecular mechanism of SSCE and its constituents are required before it is used as a preventive and/or therapeutic strategy for neurological disorders, such as HD-like syndromes.

## Author contributions

EK performed the behavioral experiments, immunohistochemistry, and Western blots analysis, and prepared the figures. MJ carried out apoptotic analysis and contributed to data interpretation. ML and JC assisted with behavioral experiments and animal maintenance. SK and DJ analyzed and commented the results. IC conceived all experiments, analyzed the results, and wrote the manuscript. All authors have read and approved the final manuscript.

### Conflict of interest statement

The authors declare that the research was conducted in the absence of any commercial or financial relationships that could be construed as a potential conflict of interest. The reviewer EC and handling Editor declared their shared affiliation.

## Supplementary material

Supplementary Data 1 No specific toxicity of SSCE is evident in the physiology of normal mice. **(A–P)** Normal mice were treated with 16, 80, 400, and 2,000 mg/kg/day of SSCE for 15 days. The body weight **(A)**, feed intake **(B)**, and water intake **(C)** were daily measured. At the end of experiment, liver **(D–H)** and kidney **(I–M)** were investigated histopathologically and mean serum levels of AST **(N)**, ALT **(O)**, and LDH **(P)** were measured by measured using enzymatic or ultraviolet assays. CV, central vein; RC, renal corpuscles. Scale bar = 100 μm.
