# Supplementary figures and images for: Schisandra chinensis Stem Ameliorates 3-Nitropropionic Acid-Induced Striatal Toxicity via Activation of the Nrf2 Pathway and Inhibition of the MAPKs and NF-κB Pathways
Source: Front Pharmacol. 2017 Sep 29;8:673. doi: 10.3389/fphar.2017.00673 (PMC5627181; doi:10.3389/fphar.2017.00673)

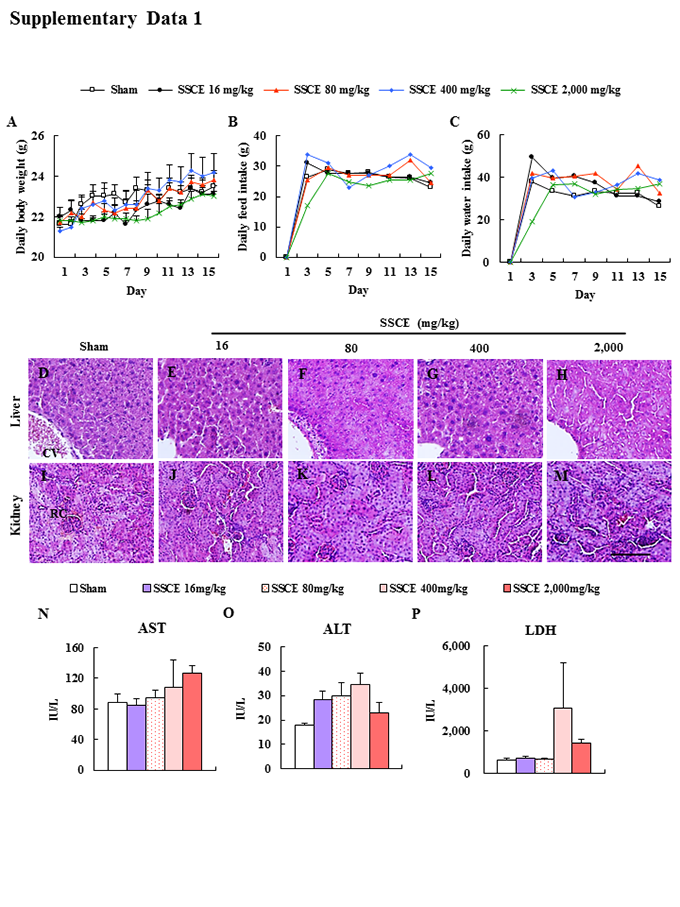

Supplement: Supplementary Data 1 — No specific toxicity of SSCE is evident in the physiology of normal mice. (A–P) Normal mice were treated with 16, 80, 400, and 2,000 mg/kg/day of SSCE for 15 days. The body weight (A), feed intake (B), and water intake (C) were daily measured. At the end of experiment, liver (D–H) and kidney (I–M) were investigated histopathologically and mean serum levels of AST (N), ALT (O), and LDH (P) were measured by measured using enzymatic or ultraviolet assays. CV, central vein; RC, renal corpuscles. Scale bar = 100 μm. [file Image1.tif]
